# 

**Published:** 1875-06

**Authors:** 


Vol. V. JUNE, 1875. No. 6.

### THE SOUTHERN MEDICAL RECORD:

S.G.O. INDEXED

PUBLISHED MONTHLY AT

ATLANTA, GEORGIA.

LOCAL EDITORS:

THOS. S. POWELL, M.D.

W. T. GOLDSMITH, M.D. R. C. WORD, M.D.

ASSOCIATE EDITORS:

| | |
|---|---|
| T. CURTIS SMITH, M.D., - - - - | MIDDLEPORT, OHIO. |
| SWAN M. BURNETT, M.D., - - - | KNOXVILLE, TENNESSEE. |
| ALBAN S PAYNE M.D., - - - - | MARKHAM'S, VIRGINIA. |
| A. R. KILPATRICK, M.D., - - - | NAVASOTA, TEXAS. |
| A. A. LYON, M D., - - - - | COLUMBUS, MISSISSIPPI. |
| G. A. BAXTER, M.D., - - - | CHATTANOOGA, TENN. |
| J. B. MATTISON, M.D., - - - - | CHESTER, NEW JERSEY. |

*"QUICQUID PRÆCIPIES ESTO BREVIS."*

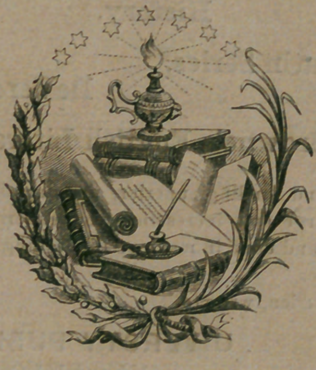

ATLANTA, GEORGIA:
SOUTHERN PUBLISHING COMPANY, PRINTERS.
1875.

TERMS, $3 00 PER ANNUM, IN ADVANCE. SINGLE NUMBER, 35 CENTS.

